# A temperature‐controlled cooling system for accurate quantitative post‐mortem MRI

**DOI:** 10.1002/mrm.29816

**Published:** 2023-08-02

**Authors:** Sebastian W. Rieger, Aaron Hess, Yang Ji, Christopher T. Rodgers, Peter Jezzard, Karla L. Miller, Wenchuan Wu

**Affiliations:** ^1^ Wellcome Centre for Integrative Neuroimaging, Oxford Centre for Human Brain Activity, Department of Psychiatry University of Oxford Oxford UK; ^2^ Wellcome Centre for Integrative Neuroimaging, FMRIB, Nuffield Department of Clinical Neurosciences University of Oxford Oxford UK; ^3^ Wolfson Brain Imaging Centre, Department of Clinical Neurosciences University of Cambridge Cambridge UK

**Keywords:** MRI, post‐mortem, quantitative imaging, temperature, unfixed

## Abstract

**Purpose:**

To develop a temperature‐controlled cooling system to facilitate accurate quantitative post‐mortem MRI and enable scanning of unfixed tissue.

**Methods:**

A water cooling system was built and integrated with a 7T scanner to minimize temperature drift during MRI scans. The system was optimized for operational convenience and rapid deployment to ensure efficient workflow, which is critical for scanning unfixed post‐mortem samples. The performance of the system was evaluated using a 7‐h diffusion MRI protocol at 7T with a porcine tissue sample. Quantitative T_1_, T_2_, and ADC maps were interspersed with the diffusion scans at seven different time points to investigate the temperature dependence of MRI tissue parameters. The impact of temperature changes on biophysical model fitting of diffusion MRI data was investigated using simulation.

**Results:**

Tissue T_1_, T_2_, and ADC values remained stable throughout the diffusion MRI scan using the developed cooling system, but varied substantially using a conventional scan setup without temperature control. The cooling system enabled accurate estimation of biophysical model parameters by stabilizing the tissue temperature throughout the diffusion scan, while the conventional setup showed evidence of significantly biased estimation.

**Conclusion:**

A temperature‐controlled cooling system was developed to tackle the challenge of heating in post‐mortem imaging, which shows potential to improve the accuracy and reliability of quantitative post‐mortem imaging and enables long scans of unfixed tissue.

## 1 INTRODUCTION

Post‐mortem MRI can bridge the complementary information provided by in vivo MRI and post‐mortem histopathology.^1^ This has potential to elucidate the neurobiological underpinnings of in vivo MRI findings, which is crucial for the development of biomarkers for disease. Post‐mortem MRI is neither limited by scan time constraints nor motion artifacts, thereby allowing for the long scan durations required to achieve high spatial resolution with high SNR, which is difficult or impossible to achieve in vivo. Moreover, ultra‐high‐field MRI allows higher SNR without increasing the scan time, which has been used in several recent studies for post‐mortem MRI at high spatial resolution.^2^, ^3^

However, a chief consideration in MRI scans is the energy transmitted by the RF pulses, which is absorbed by the sample and causes tissue heating. In vivo, this heat is dissipated by the body's thermoregulatory mechanisms (e.g., blood vessel dilation, perfusion), thus mitigating localized heating effects and regulating overall body temperature. These processes help maintain the temperatures increases due to RF pulse exposure to be kept below regulatory guidelines (i.e., < 1°C according to International Electrotechnical Commission 60601‐2‐23:2011). However, in post‐mortem MRI, the absence of thermoregulation can lead to a substantial temperature increase in the tissue, particularly during long scans at high specific absorption rate (SAR). These are precisely the conditions required for high‐resolution imaging. In addition, where the sample temperature is initially lower than ambient temperature, the problem of temperature‐related drift will be exacerbated. This would typically be the case for unfixed samples that are stored at low temperatures.

Tissue properties, such as relaxation times and diffusivity coefficients, are temperature dependent.^4^, ^5^, ^6^ For high‐resolution post‐mortem imaging, the temperature can change significantly over extended scan times, which will prevent accurate quantitative measurements of tissue properties. For unfixed samples, an additional problem is that increased temperature can speed up tissue changes and bacterial growth, as well as enzymatic function,^7^ which can confound subsequent histology.

In this work, we present a temperature‐controlled cooling system to facilitate accurate quantitative post‐mortem MRI by minimizing temperature drift during MRI scans. The developed system maintains the sample at a sufficiently low temperature to delay tissue decomposition even if scan times are long. A primary target imaging modality of the developed cooling system is diffusion MRI (dMRI), which presents significant SAR challenge at 7T and involves heavy use of gradient coils, leading to two different sources of heating: RF energy deposition and ambient heating. We present the design of the system and evaluate its performance using a 7‐h‐long dMRI scan of a porcine tissue sample at 7T. Interspersed quantitative mapping of T_1_, T_2_, and ADC at seven different time points was carried out to better understand how the diffusion measurements are likely to be affected by drift in MRI tissue parameters. The impact of temperature changes on biophysical model fitting of dMRI data was also investigated using Neurite Orientation and Dispersion Density Imaging (NODDI) model. We hypothesized that our cooling system would allow accurate estimation of biophysical model parameters by stabilizing the tissue temperature throughout the diffusion scan, while a conventional setup could suffer from significantly biased estimation.

## 2 METHODS

### 2.1 Temperature controlled cooling system

**FIGURE 1:**
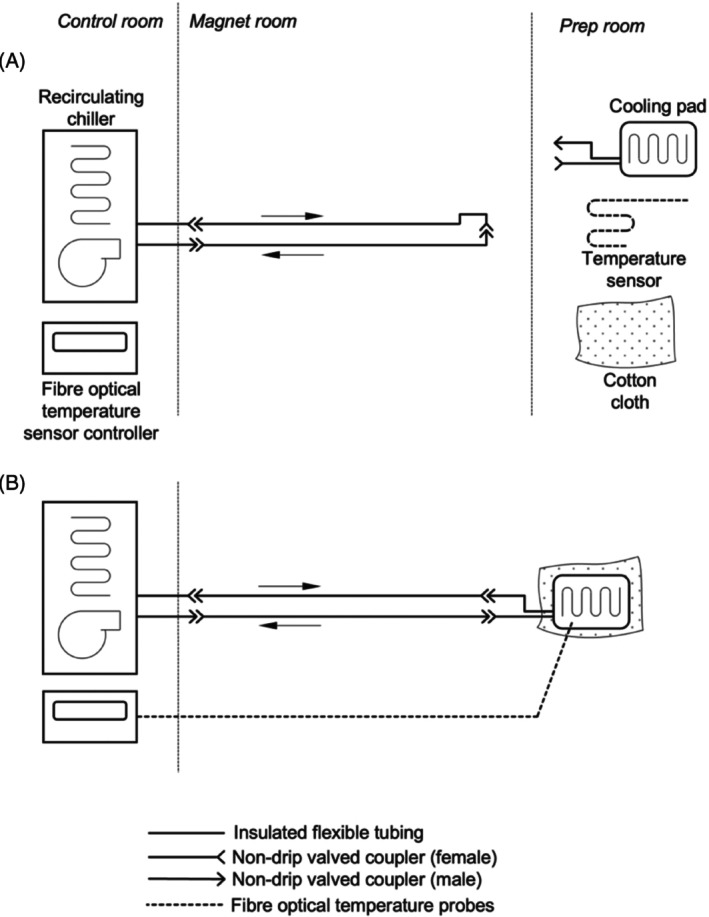
Overview of the developed temperature‐controlled cooling system. (A) Pre‐cooling and setup configuration. The chiller is operating and pre‐cooling the bulk of the fluid to the desired temperature, while the cooling pad and temperature sensor(s) (if used) are available for sample preparation in a separate preparation room. This entails affixing the temperature probes to the sample for temperature monitoring, wrapping the cooling pad around the sample for temperature control, and wrapping a layer of cloth around the outside to reduce condensation. (B) Cooling operating configuration. The prepared sample is placed in the scanner with the cooling pad connected to the chiller and temperature sensors connected to the controller. The sample temperature is monitored in real time with a sensor controller in the control room.

The developed method is based on water cooling. As shown in Figure 1, the cooling system includes a flexible cooling pad (Plastipad Infant CSZ‐193, Cincinnati Sub‐Zero, USA) that is in direct contact with the sample and a temperature‐controlled water chiller (F250, JULABO GmbH, Germany) that circulates water during scanning. The chiller has an operating range of 0 to 40°C (−10 to 40°C if antifreeze additive is used) and a temperature stability of ±0.5°C around the set point, according to the product specification. The cooling pad is wrapped closely around the sample to ensure efficient thermal contact. The pad is connected to the chiller using PVC tubing (RS PRO 914‐ 5521, RS components Ltd., UK). The tubing is insulated with closed cell foam (AF‐CO‐09X015/E‐15, Armacell, UK) to prevent excessive formation of condensation when the water temperature is lower than ambient temperature, and to ensure the temperature of the water does not change significantly as it travels from the chiller to the pad. During the scan, the post‐mortem sample and cooling pad are additionally wrapped in an outer layer of cotton cloth to prevent formation of condensation on the surface of the cooling pad from the moisture in the ambient air.

The system is optimized for operational convenience and rapid deployment, ensuring efficient workflow that is critical for scanning unfixed post‐mortem samples. Within the recruitment model of the study for which this system was designed, deployment has to be possible with very little notice. The system comprises three parts. The first part includes the chiller placed in the control room with short lengths of insulated PVC tubing, which pass through a waveguide in the penetration panel between the control room and magnet room. The small footprint (240 × 400mm) of the chiller enables it to be permanently left in place even within a relatively small control room. The second part is a set of insulated extension tubing of suitable length to reach from the penetration panel to the magnet isocenter. The third component is the cooling pad, attached to which are two short lengths of insulated tubing using push‐on hose unions (RS PRO 795‐354, RS Components Ltd., UK) and nylon snap grips (RS PRO 291‐616, RS Components Ltd., UK). The second and third parts are easily portable, meaning they can be stored conveniently on site and brought into the imaging suite when needed. Connection between the three parts is made using pairs of non‐spill valved hose couplings (type NS4D220‐06 (male) and NS4D170‐06 (female), Colder Products Company, USA), allowing the making and breaking of connections without spillage, and storage of the extension hoses and cooling pad pre‐filled and ready to use. In the pre‐cooling configuration, a cooling circuit can be formed without the pad present (Figure 1A). This improves the workflow efficiency by allowing two procedures to happen simultaneously: namely sample preparation with the pad, and pre‐cooling of the bulk of the liquid in the chiller reservoir and in the extension tubing. Pre‐cooling may also take place while the scanner is still being used for a preceding experiment. Once the sample is ready, it can be placed in the scanner and connected to the temperature control system which is then already at operating temperature. Figure S1 shows a picture of all components of the system.

One potential issue in using a water‐cooled system is that circulating water may appear in the MRI images. This signal can be removed with a suitable mask in post‐processing for some MRI modalities. However, for EPI‐based acquisition, which is commonly used for dMRI acquisition, the circulating water signal might confound the tissue signal due to distortion and/or signal aliasing into the tissue of interest. We resolved this by using a 5 mM aqueous solution of manganese chloride as the coolant fluid, which has a very short T_2_ (3–5ms)^8^ and therefore is invisible in dMRI images which are commonly acquired with a long TE (typically 40–80 ms). Due to the low concentration of manganese chloride in the cooling fluid, its heat capacity is assumed to remain similar to that of pure water, as does the permissible operating temperature range. The lower limit for operation without antifreeze additive is 5°C as per the chiller manufacturer's specification. The flow rate in the system was 1 L/min.

To evaluate the efficacy of the temperature control system, we recorded temperatures of the sample at 0.1 Hz sampling rate using fiber optic temperature probes, which were connected to a signal conditioner. Both the chiller and the signal conditioner were sited in the MRI control room, outside of the Faraday shield and the 5 Gauss contour.

### 2.2 MRI experiment

All experiments were performed on a porcine tissue sample in a 7T whole body MRI scanner (Siemens Magnetom, Erlangen, Germany) using a custom‐built post‐mortem head coil (RAPID Biomedical) with quadrature transmit and 16 receive channels. The sample was prepared by wrapping a single 1200 g piece of fresh pork meat (a muscle section taken from a pork loin joint) tightly in cling film to maintain an approximately cylindrical shape. A target post‐mortem MRI experiment of interest is high spatial‐ and angular‐resolution dMRI, which is challenging due to long scan times (7 h for the target protocol), significant SAR at 7T (especially with multi‐band excitation), and heavy use of gradient coils. The dMRI scan was interrupted periodically at an averaged interval of 78 min (all intervals: 78, 76, 77, 78, 81, 78 min) to measure a set of quantitative maps of T_1_, T_2_, and ADC in 6 min 20 s. Seven sets of quantitative maps were acquired, leading to a total scan time of 8 h. Two MRI experiments were conducted in a single day using the same acquisition protocols but with different cooling configurations: the first experiment with the cooling system turned on (cooling‐on) and the second with the cooling system turned off (cooling‐off). For the cooling‐on experiment, the operating temperature of the chiller was set to 8°C. The chiller took about 30 min to cool the system including the bulk of the liquid from room temperature (21°C) to the operating temperature. A 4‐h gap was inserted between the two experiments with cooling on to reset the temperature of the sample.

#### 2.2.1 Diffusion MRI protocol

The protocol was designed for acquiring high spatial‐ and angular‐resolution dMRI data from unfixed ex vivo neonatal brain in situ, which uses a 2D simultaneous multi‐slice spin‐echo readout‐segmented EPI sequence with monopolar diffusion preparation.^9^, ^10^ Acquisition parameters were: FOV = 150 × 150 mm, matrix = 188 × 188, 130 slices, 0.8 mm isotropic resolution, GRAPPA acceleration factor = 2, multi‐band factor = 2, TR = 10.8 s, TE = 113 ms, seven readout segments. A total of 280 diffusion‐weighed volumes were acquired for three *b* values of 3000, 6000, and 9000 s/mm^2^ with unique diffusion encoding directions (64, 88, and 128 directions, respectively) together with 21 *b* = 0 volumes. The acquisition time for the dMRI protocol was just under 7 h.

#### 2.2.2 Quantitative mapping protocols

Quantitative T_1_ maps were acquired using a saturation‐recovery single‐shot acquisition (SASHA),^11^, ^12^ which uses an image with no saturation and seven images with pre‐saturation delay times of 0, 300, 400, 500, 600, 1100, 1600 ms. Acquisition parameters: FOV = 150 × 150 mm, resolution = 1.2 × 1.2 mm, five slices with 5 mm slice thickness and 20 mm slice gap, acquisition time = 1 min 35 s. Quantitative T_2_ maps were measured using a multi‐echo spin‐echo sequence using the same FOV and resolution as the T_1_ mapping protocol. Eight echoes were acquired at TEs = 10/20/30/40/50/60/70/80 ms, and TR = 2500 ms. The acquisition time was 2 min 49 s.

ADC maps were acquired with 6 *b* = 1000 s/mm^2^ volumes with non‐coplanar diffusion directions and one *b* = 0 volume. Imaging used a 2D diffusion‐weighted readout‐segmented EPI sequence, FOV = 150 × 150 mm, 5 readout segments, GRAPPA factor = 3, echo spacing = 0.38 ms, 21 slices with 5 mm slice thickness and no slice gap, 1.2 mm in‐plane resolution, acquisition time = 1 min 56 s.

For one complete set of T_1_, T_2_, and ADC maps, the scan time was 6 min 20 s. The total scan time of the full protocol, including the diffusion acquisition and seven measurements of quantitative maps, was ˜8 h.

### 2.3 Temperature recording

Temperatures near the center and at the surface of the sample were recorded during the MRI experiments. The settings for ambient air conditioning, heating, and scanner bore fan speed were kept identical for both the cooling‐on and cooling‐off experiments.

### 2.4 Experimental data analysis

T_1_ maps were calculated with pixelwise R1 fitting as described in Reference 11. T_2_ maps were calculated using Siemens MapIt software (Siemens Medical Solutions, Erlangen, Germany). The diffusion MRI data were pre‐processed with FSL's eddy_correct, followed by tensor fitting with FSL's dtifit tool.^13^ Mean T_1_, T_2_, and ADC values across the sample were calculated within a tissue mask covering all slices of the sample. To quantitatively assess the temperature dependence of tissue MRI parameters, a linear regression model was applied to the measured T_1_, T_2_, and ADC values obtained from the cooling‐off experiment.

### 2.5 Diffusion simulation

To investigate the predicted impact of temperature changes on biophysically modeling of dMRI data, we simulated the dMRI signal dependence on temperature. Two temperature configurations were assessed with a temperature increase of 1.98°C and 11.38°C, corresponding to the measured temperature changes at the sample center with cooling on and off, respectively. T_1_ and T_2_ values at a given temperature were simulated based on linear models fitted using the T_1_ and T_2_ maps measured from the sample. NODDI model was used to simulate diffusion weighting and fit the simulated data. Simulation details are included in Supporting Information.

## 3 RESULTS

**FIGURE 2:**
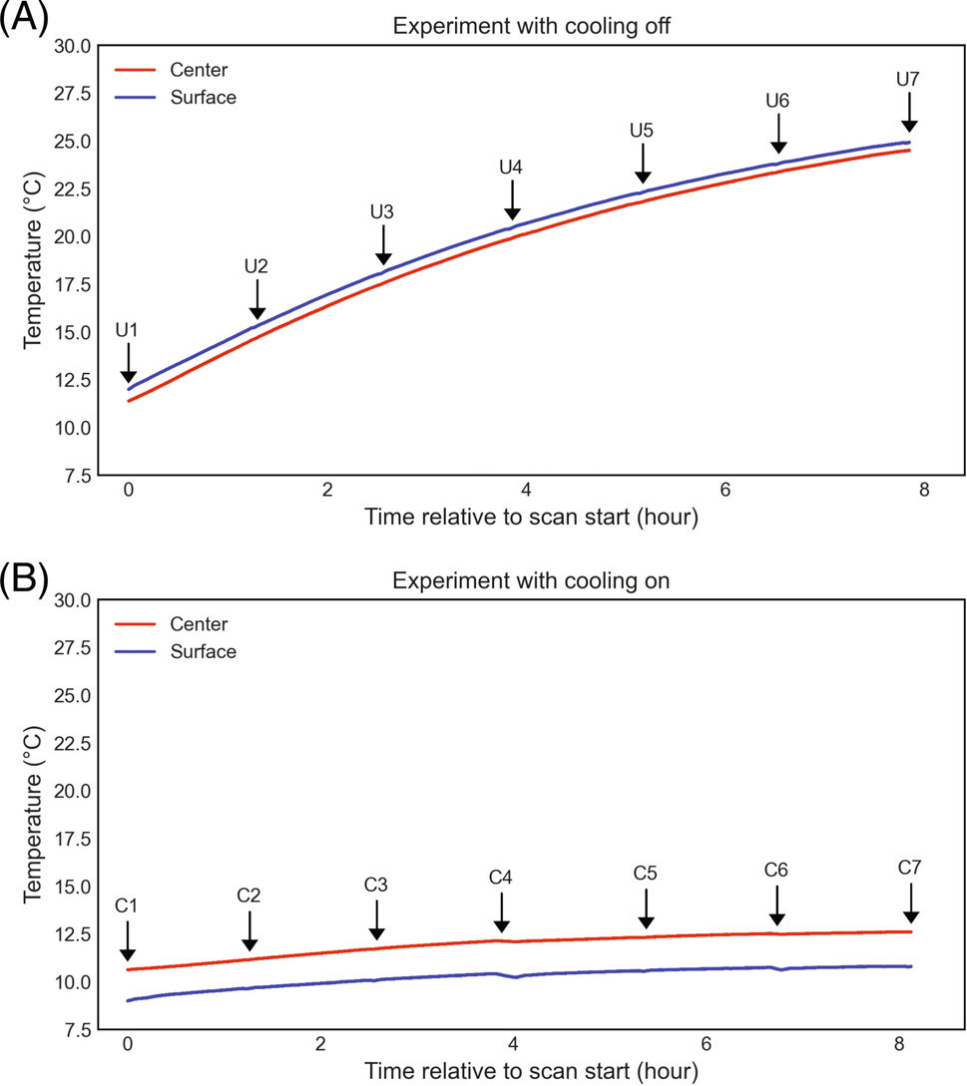
Sample temperature measured during MRI experiments with cooling system (A) turned off and (B) turned on. The same MRI protocols were used in the two scans. The initial temperatures were comparable between the two samples prior to both scans. T_1_, T_2_, and ADC maps were acquired at interspersed time points during the diffusion acquisition, with an average time interval of 78 min. These time points were indicated with ‘C#’ for scanning with cooling on and ‘U#’ for scanning with cooling off, respectively. Temperatures were recorded at a rate of 0.1 Hz using two fiber optic temperature probes, near the center (red lines) and on the surface (blue lines) of the sample.

Figure 2 shows the temperature changes recorded during the two MRI experiments. For the first 8‐h scan (cooling on), the sample had been stored in a domestic refrigerator but warmed up during preparation, resulting in a temperature of 10.63°C at the center at the start of the experiment. The surface temperature was 8.99°C at the start of the scan, which was close to the operating temperature of the chiller (i.e., 8°C). For the second scan (cooling off), the temperature at the center and the surface were 11.38 and 11.99°C, respectively, at the start of the scan. Throughout the cooling‐on scan, the temperature at the center of the sample changed from 10.63 to 12.61°C, and at the surface from 8.99°C to 10.8°C, representing an increase of 1.98 and 1.81°C, respectively. For the cooling‐off scan, the temperature at the center of the sample rose from 11.38 to 24.5°C and the surface temperature from 11.99 to 24.92°C, representing an increase of 13.12 and 12.93°C, respectively. These results suggest that the temperature‐controlled cooling system can stabilize the temperature of the sample during MRI scans of extended duration. During the cooling‐on experiment, the temperature at the sample surface, which is close to the cooling pad, remained below the sample center temperature. This is inverted in the cooling‐off experiment where the sample surface warmed up more quickly than the center, likely due to its adjacency to ambient air that is above the sample temperature. It is worth noting that the starting temperatures of the sample, which were designed for post‐mortem scanning of unfixed tissue where a low temperature is required to delay tissue degradation, were well below the room temperature in these experiments. If tissue degradation is not the main concern (e.g., scanning fixed tissue), the sample can be brought to room temperature before the scan.

**FIGURE 3:**
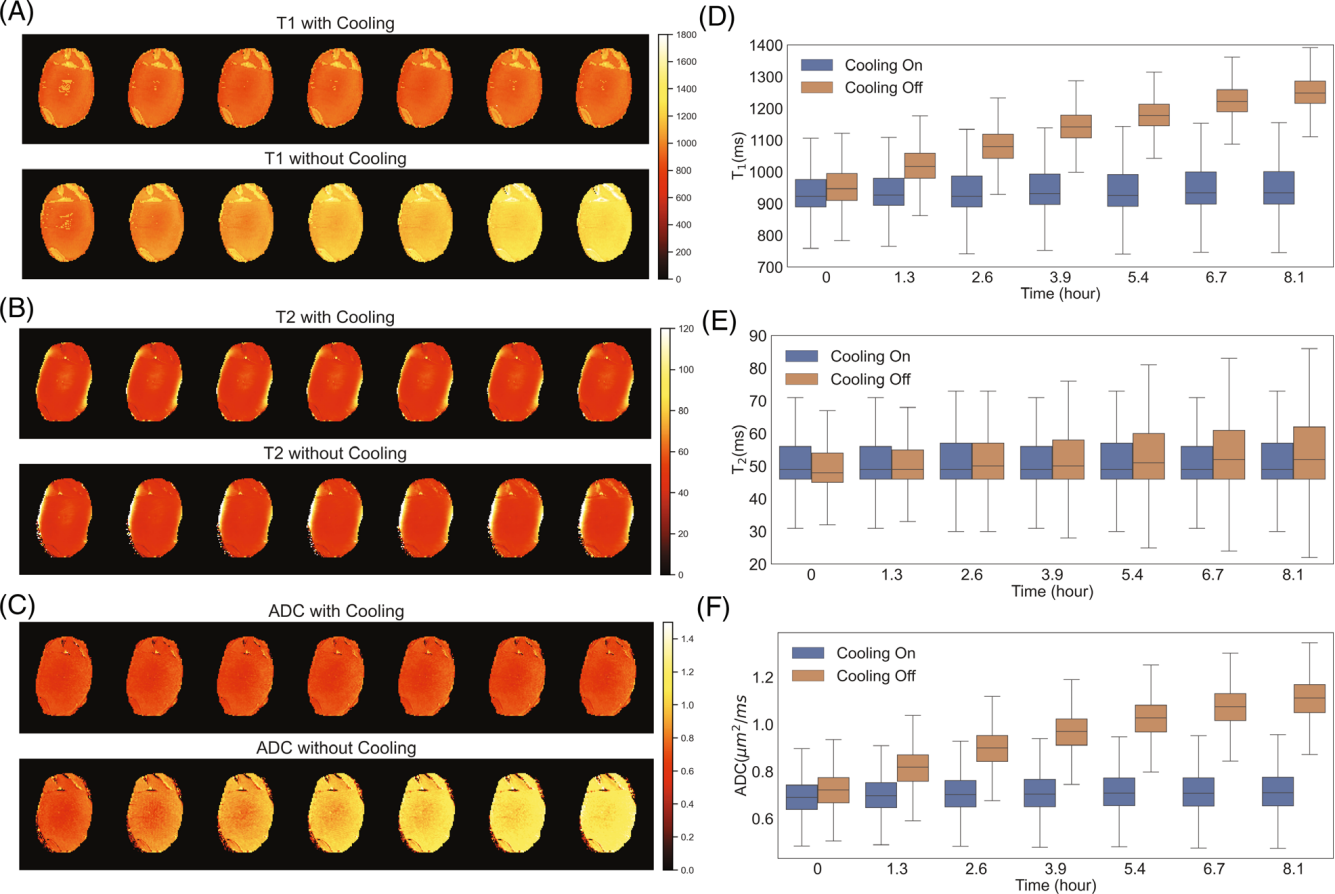
Quantitative maps of T_1_, T_2_, and ADC acquired from the two MRI experiments. Left (A‐C): quantitative maps of an axial slice acquired at seven time points. Right (D‐F): Box‐and‐whisker plot of T_1_, T_2_, and ADC values within the sample at seven time points.

The measured T_1_, T_2_, and ADC values at all seven time points are shown in Figure 3. For the cooling‐on experiment, the T_1_, T_2_, and ADC maps are highly consistent (Figure 3) across time. The mean T_1_/T_2_/ADC values are 945 ms/52 ms/0.69 μm^2^/ms at the start of the experiment, and 951 ms/54 ms/0.70 μm^2^/ms at the end. The relative changes for T_1_/T_2_/ADC over the 8 h are 0.6%/2.5%/3.6%. In cooling‐off expriment, the acquired parameter maps exhibit a larger change in T_1_ and ADC, and a moderate change in T_2_, over time. The mean T_1_/T_2_/ADC values at the beginning of scanning are very close to those from the first experiment, 922 ms/55 ms/0.69 μm^2^/ms, as one might expect given the similar initial sample temperatures. After the 8‐h scan, the mean T_1_/T_2_/ADC values increase to 1206 ms/61 ms/1.05 μm^2^/ms, due to the temperature rise. The relative changes to T_1_/T_2_/ADC are 31%/11%/52%. The results show that temperature changes during the scan can lead to strong variations in tissue MRI parameters. For high‐resolution quantitative measurement that require long scan times, this will lead to biased estimation of quantitative parameters. In contrast, stable T_1_, T_2_, and ADC values can be achieved throughout extended scan durations using the developed temperature‐controlled cooling system, despite the significant heating challenge.

**FIGURE 4:**
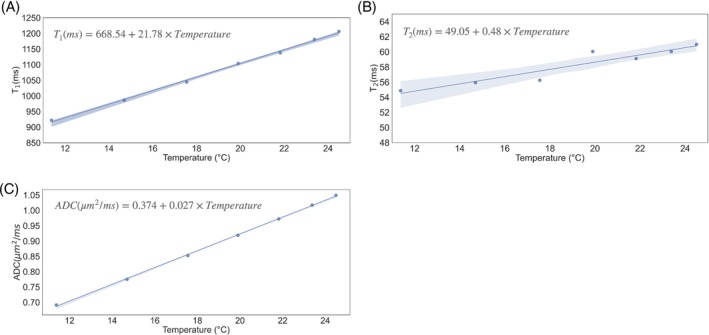
Temperature dependence of tissue parameters. Scatter plots of measured (A) T_1_, (B) T_2_, and (C) ADC values against temperature measured at the center of the sample for the second scan, which was performed with cooling off. Results of the linear regression fit are shown for each parameter. The *R* ^2^ and *p*‐value of the linear regression fitting are: T_1_(*R* ^2^ = 0.998, *p* < 0.0001), T_2_(*R* ^2^ = 0.888, *p* < 0.001), ADC(*R* ^2^ = 0.999, *p* < 0.0001).

From the data of the second scan (cooling off), there was a statistically significant and strong correlation of the linear regression model for the measured T_1_, T_2_, and ADC and temperature (*p* < 0.05) (Figure 4). The slope in percentage parameter can be calculated as the slope divided by the parameter value at the start of the scan, which gives the rate of relative change of each tissue parameter compared to the starting point. ADC (3.97% per °C) has the strongest slope in percentage units from the linear model fit, followed by T_1_ (2.36% per °C). The slope in percentage for T_2_ is relatively modest (0.87% per °C).

**FIGURE 5:**
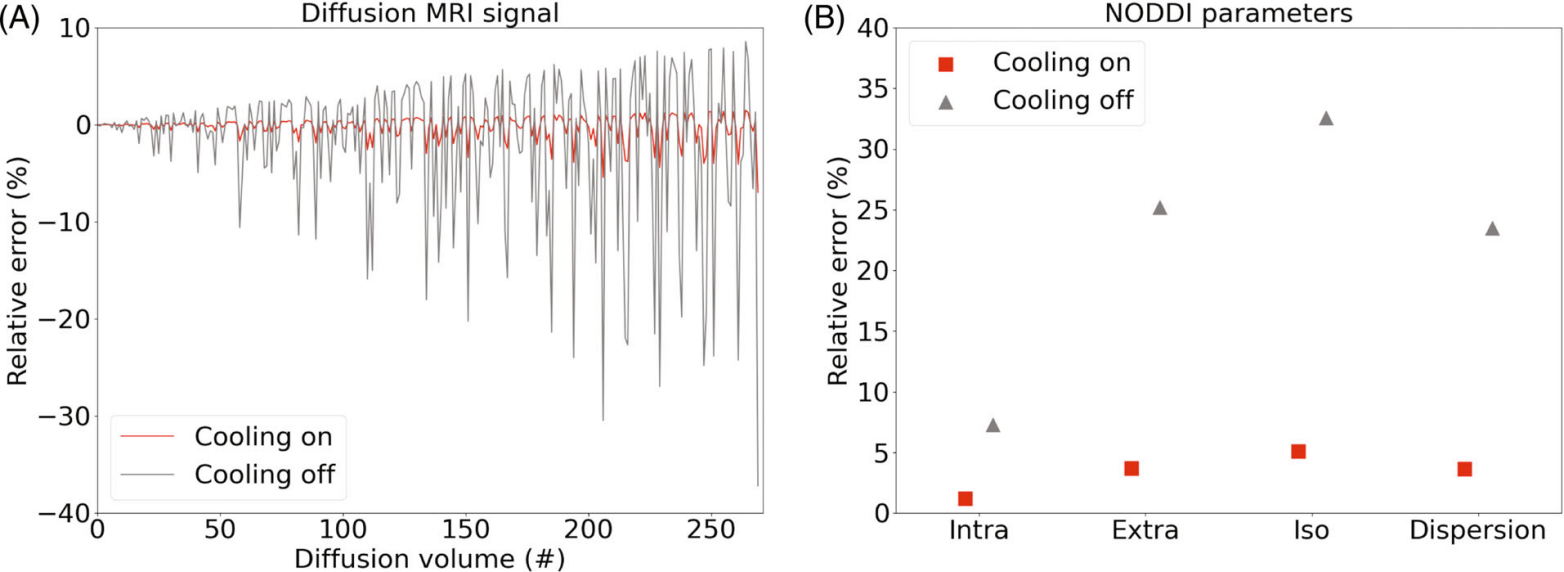
Impact of temperature changes during a dMRI scan on signal intensity and NODDI model fitting. Two dMRI datasets were simulated with temperature increases of 1.98 and 13.12°C, corresponding to the temperature changes during the experiment with cooling on and cooling off, respectively. (A) The relative errors (in percentage unit) for all diffusion volumes compared to the reference, which was simulated with constant temperature. (B) The relative error (in percentage unit) of NODDI parameters fitted from the two dMRI datasets comparing to the ground truth, including intra‐cellular volume fraction (‘Intra’), extra‐cellular volume fraction (‘Extra’), isotropic volume fraction (‘Iso’), and axon orientation dispersion (‘Dispersion’). Note the spikes in (A) are due to certain diffusion gradient directions being aligned with the assumed mean axon orientation in the NODDI model, which results in a low dMRI signal and thus a higher relative error.

Figure 5 shows the diffusion MRI simulation results. Due to the temperature dependence of T_1_, T_2_, and water diffusivity, temperature changes cause dMRI signal to deviate from the reference, which corresponds to the dMRI signal with constant temperature (Figure 5A). The relative errors of dMRI signal are much higher for the cooling‐off simulation (e.g., >30% for some later volumes) than cooling‐on simulation (e.g., <5% for most volumes). In NODDI parameter fitting, the relative errors with the cooling‐off simulation are higher than that with cooling‐on: intra‐cellular volume fraction (7.2% vs. 1.2%), extra‐cellular volume fraction (25.2% vs. 3.7%), isotropic volume fraction (32.6% vs. 5.1%), and axon orientation dispersion index (23.5% vs. 3.6%).

## 4 DISCUSSION AND CONCLUSION

In this work, we developed a temperature‐controlled cooling system to tackle the challenge of heating in post‐mortem imaging. This system aims to enable accurate quantitative post‐mortem MRI scans by maintaining a stable, low temperature of the sample over long scan times. The MRI experiment performed with cooling off represents a scenario one might face with a long post‐mortem scan, for example aiming to achieve high spatial resolution, where the sample temperature will rise rapidly during the scan. As demonstrated in Figures 3 and 4, this will lead to temporally varying tissue parameters and bias quantitative post‐mortem measurement. In contrast, we have demonstrated that our cooling system could provide a stable sample temperature and consistent quantitative measures across time (Figure 3). The results demonstrate the efficacy of the developed cooling system and its potential to improve the accuracy and reliability of quantitative post‐mortem imaging.

We considered both air‐ and water‐based cooling. Air cooling was initially expected to be preferable because it does not produce any signal in the image that might be confounded with the tissue signal. We built a prototype air‐cooling system fed from compressed air cylinders, cooling the air to approximately 0°C using heat exchangers in an ice bath. This system was tested on a cylindrical meat phantom and found to be effective at maintaining a low sample temperature. However, the required flow rate would have made the use of air cylinders impractical for longer scans. Replacing them with a suitable air blower or compressor was judged to be impracticable, owing to the cost, space requirement, and acoustic noise emission of such a device. Therefore, our final design used water cooling.

Previously, Birkl et al.^15^ developed a temperature control system, which used a temperature‐controlled water bath to control the temperature of tissue samples that were embedded in agar gel and vacuum sealed. This system is particularly suited to small samples (e.g., extracted brain slices), which can be vacuum sealed and placed inside a fluid filled container for scanning. It is able to maintain the sample temperature to the desired value with a small variation (0.5°C) but cannot operate continuously during scanning because it causes flow artifacts from water. A potential solution to this problem could be to use the 5 mM aqueous solution of manganese chloride instead of water, as used in our setup. However, for larger samples that would not conveniently fit in a suitable container, or samples that cannot be vacuum packed and/or submerged in the cooling fluid (as is the case in our neonate scans), circulating the fluid through a cooling pad wrapped around the sample, as described in our paper, would be the better option.

The impact of temperature on quantitative post‐mortem imaging has been studied before, albeit with a focus on how temperature differences introduce discrepancy of tissue parameter estimates between in vivo and post‐mortem scans.^14^, ^15^, ^16^, ^17^ These studies used short scan times and assumed a stable temperature during the acquisition of each quantitative map. However, under certain conditions, such as long scan durations, low initial sample temperatures, and the use of high SAR pulses, our results demonstrate that this assumption may not be valid and quantitative measures from the post‐mortem data become biased. Even if temperatures do not drift during an experiment, the amount of bias will depend on both the initial temperature of the sample and ambient temperature of the scanner bore, confounding reproducibility.

Temperature variations in post‐mortem MRI are particularly challenging for quantitative imaging due to the longer scan time needed to acquire multiple image volumes which are weighted toward different contrasts. Here, we studied diffusion biophysical model fitting using simulations of temporally varying temperatures with the NODDI model. The results suggest that the estimation of NODDI parameters can be strongly biased if the temperature change is large due to the temperature dependence of T_1_, T_2_, and water diffusivity. The impact on NODDI mean orientation estimation is very small (less than 1°, not shown), which agrees with the recent study by Lenz et al.,^16^ which shows that fiber angle maps from DTI fitting are comparable between in vivo and post‐mortem brains despite different tissue temperatures. Using the developed cooling system, the temperature rise during the diffusion scan is less than 2°C (Figure 2B), enabling a more accurate estimation of the NODDI parameters. In comparison, without cooling, the temperature change would exceed 2°C in less than 1 h (Figure 2A). If the scan time were restricted to such a value, this would limit the achievable spatial resolution if one wanted to achieve an equivalent error level as with cooling on.

The multi‐echo spin echo sequence used for T_2_ mapping is prone to contamination with stimulated and indirect echoes, which may lead to inaccurate T_2_ values. However, as our main interest in this work is the change of T_2_ with temperature, which we expect to be still detectable in the presence of imperfect T_2_ measurements, and so we believe this would not impact our primary finding.

The developed cooling system can not only maintain a stable sample temperature during the post‐mortem scan but can also maintain a low sample temperature (˜10°C in our experiment) as is required to maintain the integrity of unfixed tissues. The resulting temperature differential between the tissue and ambient air will itself lead to some tissue warming even in the absence of SAR or gradient‐induced heating. Without temperature stabilization, this places a limit on scan times, and therefore on the achievable resolution and SNR. With the cooling system presented here, it is possible to scan post‐mortem tissue while keeping it at low temperatures for many hours, enabling more ambitious and reproducible post‐mortem imaging experiments. The developed system may also be used with tissue samples kept in liquid (e.g., phosphate‐buffered saline), although the efficacy of the cooling needs to be assessed. In addition, by replacing the chiller with a refrigerated/heating circulator (e.g., model CD‐200F, JULABO GmbH, Germany), the developed system may also be used to stabilize tissue sample temperatures at a value above ambient temperature if needed.

With the cooling system turned on, the temperature of the sample would gradually change towards a steady state. The final temperature depends on multiple factors, including the temperature and flow rate of the fluid, the thermal coupling between the pad of the sample, and the heat absorbed by the sample during the scan. In our experiment, the steady state temperature was approximately 2°C higher than the starting temperature. This temperature rise could be potentially reduced using a more sophisticated cooling system design with closed loop feedback control that can adjust the flow rate and/or the temperature of the fluid based on the real‐time temperature data measured from the sample.

Note there is a ˜1.7°C difference between the surface and center temperatures for the cooling‐on scan, which is because heat was generated throughout the sample, while the cooling system only acted upon the sample surface, making it inevitable that a temperature gradient develops to allow heat to be transferred to the surface. In the cooling‐off condition, the difference between the surface and the center (˜0.5°C) was smaller as heat was not efficiently removed from the surface, and surface temperature rose as overall sample temperature rose.

For the cooling‐off experiment, which was performed second, the scan started when the temperature of the sample center reduced to a similar value as the starting temperature of the cooling‐on experiment. Although the starting temperatures of cooling‐on and cooling‐off experiments were not exactly the same, our main finding is still valid, which shows the ability of our system to main a stable sample temperature.

The temperature control system was initially developed for in situ, ex vivo neonatal brain MRI. Due to the sensitive nature of this type of study, every effort must be made to prevent damage to the neonatal tissue. Accordingly, it is not possible to measure the temperature inside of the neonatal brain to accurately evaluate the performance of the cooling system. It was therefore decided to use a cylindrical pork tissue sample for validating and characterizing the system. The temperature sensors were placed at the surface and in the center of the sample (which would not be possible once the system would be used for in situ, ex vivo neonate scan) and we characterized temperature changes during prolonged high‐SAR scanning. For scanning of extracted brain samples, there might be fewer limitations to measuring the temperature of the sample directly, which can provide a more accurate evaluation of the performance of the developed system.

In this work, we only evaluated the performance of the cooling system on a porcine tissue sample with a size of approximate 18 cm in length and 9 cm in diameter. The shape and dimensions of the porcine sample were chosen to approximate those of samples this system was originally designed for (i.e., ex vivo, in situ neonate brains). For any sample of similar or smaller size, the temperature change is expected to remain within 2°C if using the cooling system with the same setup (i.e., scan protocols and field strength) as in this work. However, to apply the system to samples with a larger size and/or very different shape, or a different scan setup that may lead to higher heat deposition (e.g., using sequences with a higher SAR profile, field strength higher than 7T, and scan durations longer than 7 h), it is necessary to run a similar experiment as we did in this work to evaluate the cooling performance to ensure a uniform cooling throughout the experiment. If the current setup could not provide sufficient cooling in certain conditions, approaches including increasing the flow rate of the coolant fluid, improving thermal coupling between the pad and the sample, or a combination of both can be used to improve the cooling efficiency.

A major goal of developing the cooling system is to preserve the tissue integrity by maintaining a low temperature to avoid significant tissue degeneration. This is important, for example, if the tissue is to be used for histological analysis after the post‐mortem MRI scan. In our study, ethical approval required tissue condition to be unaltered during the scan due to the sensitive nature of the study. However, if preserving tissue integrity is not critical, scanning at body temperature would allow more consistent tissue parameter values between ex vivo and in vivo, which may benefit data interpretation and analysis. In this work, the start temperatures of the experiments were chosen to match the starting temperature (˜10°C) of in situ, ex vivo neonatal brain MRI scans we had conducted. The relative errors of dMRI signal and NODDI parameter estimation may be reduced by starting from body temperature instead of a lower temperature as tested in this work. This is because the relative diffusivity change compared to the diffusivity at the start of the scan is smaller at higher temperatures (Figure S2), which leads to smaller fitting errors. However, the impact of temperature change on quantitative parameter measures still exists even when scanning at body temperature. Hence, it is still recommended to use the developed cooling system to maintain a constant temperature.

## CONFLICT OF INTEREST STATEMENT

P.J. is the Editor‐in‐Chief of Magnetic Resonance in Medicine. In line with COPE guidelines, he recused himself from all involvement in the review process of this paper, which was handled by an Associate Editor. He and the other authors have no access to the identity of the reviewers.

## Supporting information


**Data S1.** Supporting information.
