# Supplementary material for: A temperature‐controlled cooling system for accurate quantitative post‐mortem MRI
Source: Magn Reson Med. 2023 Aug 2;90(6):2643–52. doi: 10.1002/mrm.29816 (PMC10952464; doi:10.1002/mrm.29816)
Supplement: Supplementary file 1 — Data S1. Supporting information. [file MRM-90-2643-s001.docx]

**Supporting Information**

Diffusion MRI simulation details

To investigate the predicted impact of temperature changes within a diffusion MRI scan on biophysically modelling, we simulated the diffusion MRI signal dependence on temperature based on following assumptions.

First, T1 and T2 values at a given temperature were simulated based on linear models which were fitted using the T1 and T2 maps measured from the sample (see Figure 4 and Results for details). The T1 and T2 weighting on the diffusion MRI signal can be calculated as(1):

$S_{T_{1},T_{2}}=M_{0}sin\left( \theta_{ex} \right)\sin^{2}\left( \theta_{ref}/2 \right)\frac{1+\left( cos\left( \theta_{ref} \right)-1 \right)e^{-\left( TR-TE/2 \right)/{T_{1}}}-cos\left( \theta_{ref} \right)e^{-{TR}/{T_{1}}}}{1-cos\left( \theta_{ref} \right)cos\left( \theta_{ex} \right)e^{-{TR}/{T_{1}}}}e^{-{TE}/{T_{2}}}$ Eq. 1

where $\theta_{ex}$ and $\theta_{ref}$ are the angles for excitation and refocusing pulses.

Second, diffusion weighting is calculated using the NODDI method (2), which represents diffusion MRI signal with a biophysical model using parameters including axon orientation dispersion (OD) index, mean axon orientation, isotropic diffusivity (d_iso_), intrinsic parallel diffusivity (d_//_), and volume fractions for intra-cellular (f_intra_), extra-cellular (f_extra_) and isotropic (f_iso_) compartments. The isotropic diffusivity and intrinsic parallel diffusivity at a given temperature is calculated based on the Speedy-Angell power-law (3) and Arrhenius equation (4,5), respectively. Data simulation and model fitting were performed using the DMIPY package (6). The final diffusion MRI signal was calculated as the product of T1- and T2-weighted signal and the NODDI simulated signal.

Two temperature configurations were assessed with a temperature increase of 1.98°C and 11.38°C, which correspond to the measured temperature changes at the sample centre with and without the cooling system turned on, respectively. The starting temperature was set to 10.63°C as measured at the sample centre in the experiment with the cooling system turned on. Other NODDI parameters were set to OD=0.1, f_iso = 0.25, f_intra = 0.8, f_extra=0.2 and mean axon orientation= (45°, 45°) (polar and azimuthal angles). The Human Connectome Project (HCP) 3T acquisition protocol (7) was used for the simulation of three b values (1000,2000 and 3000s/mm^2^), 288 diffusion directions, TR/TE=5520/89.5ms, $\theta_{ex}/\theta_{ref}$=78°/160°.

The simulated diffusion MRI signal was fitted with the NODDI model with fixed d_iso_ and d_//_ values corresponding to the temperature at the start of the scan (i.e., 10.63°C). Relative error of the estimated NODDI parameters related to the ground truth NODDI parameters were calculated to investigate the effect of temperature change on NODDI model fitting. Relative error of the simulated dMRI signal with temperature changes related to a reference dMRI signal simulated without temperature changes was also calculated.


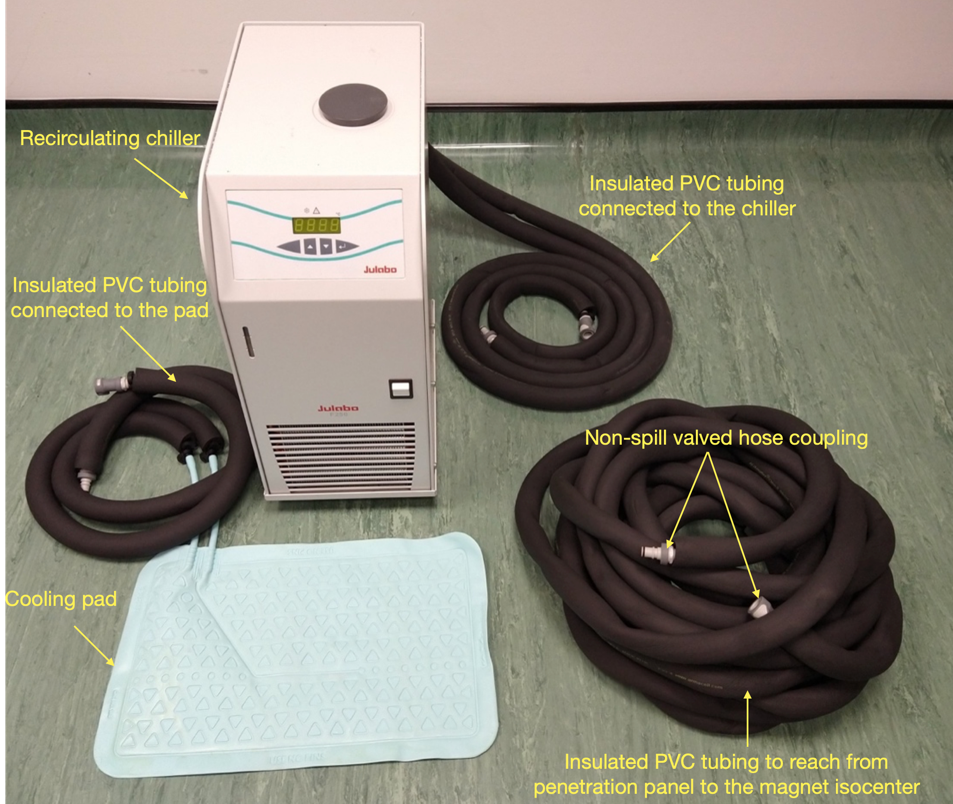


*Supplementary Information Figure S1. A picture of the developed temperature-controlled cooling system placed together.*


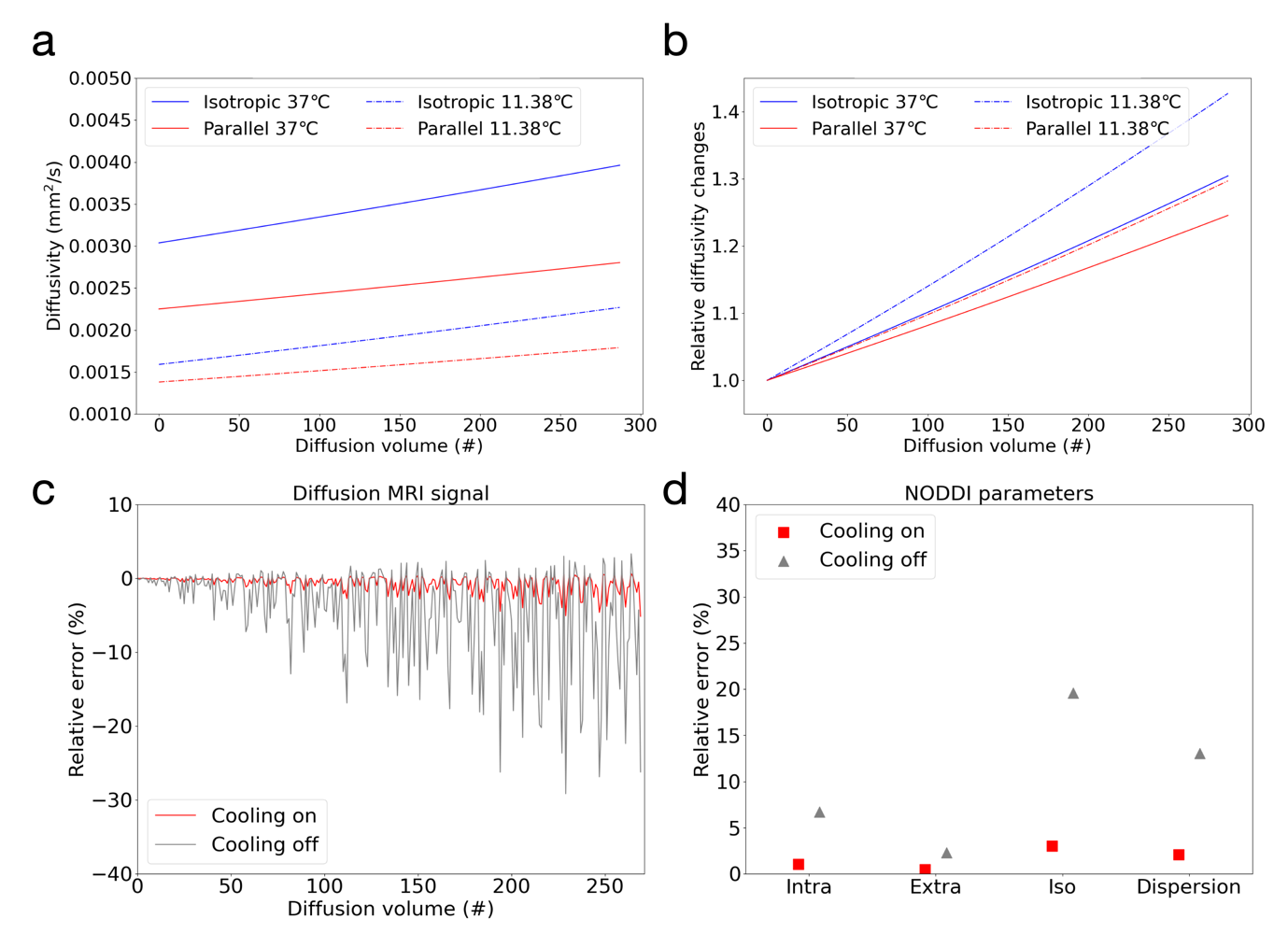


*Supplementary Information Figure S2. Absolute (a) and relative (to the starting point of the scan) (b) changes of isotropic diffusivity (‘Isotropic’) and intrinsic parallel diffusivity (‘Parallel’) as a result of temperature changes during dMRI scans. Two starting temperatures are simulated: body temperature of 37°C (solid line) and temperature measured in this study, 11.38°C (dash-dotted line). Impact of temperature changes during a dMRI scan on signal intensity (c) and NODDI model fitting (d) with starting temperature of 37°C (body temperature) are also shown. Two dMRI datasets were simulated with temperature increases of 1.98°C and 13.12°C corresponding to the temperature changes during the experiment with cooling on and cooling off, respectively, assuming the temperature changes are the same as observed at a lower starting temperature in the phantom experiments. (c). The relative errors (in percentage unit) for all diffusion volumes compared to the reference, which was simulated with constant temperature. (d). The relative error (in percentage unit) of NODDI parameters fitted from the two dMRI datasets comparing to the ground truth, including intra-cellular volume fraction (‘Intra’), extra-cellular volume fraction (‘Extra’), isotropic volume fraction (‘Iso’) and axon orientation dispersion (‘Dispersion’). Note the spikes in (c) are due to certain diffusion gradient directions being aligned with the assumed mean axon orientation in the NODDI model, which results in a low dMRI signal and thus a higher relative error.*

**Reference**

1. Engström M, Mårtensson M, Avventi E, Skare S. On the signal-to-noise ratio efficiency and slab-banding artifacts in three-dimensional multislab diffusion-weighted echo-planar imaging. Magnetic Resonance in Medicine 2015;73:718–725 doi: 10.1002/mrm.25182.

2. Zhang H, Schneider T, Wheeler-Kingshott CA, Alexander DC. NODDI: Practical in vivo neurite orientation dispersion and density imaging of the human brain. NeuroImage 2012;61:1000–1016 doi: 10.1016/j.neuroimage.2012.03.072.

3. Holz M, Heil SR, Sacco A. Temperature-dependent self-diffusion coefficients of water and six selected molecular liquids for calibration in accurate 1H NMR PFG measurements. Phys. Chem. Chem. Phys. 2000;2:4740–4742 doi: 10.1039/b005319h.

4. Dhital B, Reisert M, Kellner E, Kiselev VG. Intra-axonal diffusivity in brain white matter. NeuroImage 2019;189:543–550 doi: 10.1016/j.neuroimage.2019.01.015.

5. Dhital B, Labadie C, Stallmach F, Möller HE, Turner R. Temperature dependence of water diffusion pools in brain white matter. NeuroImage 2016;127:135–143 doi: 10.1016/j.neuroimage.2015.11.064.

6. Fick RHJ, Wassermann D, Deriche R. The Dmipy Toolbox: Diffusion MRI Multi-Compartment Modeling and Microstructure Recovery Made Easy. Frontiers in Neuroinformatics 2019;13.

7. Van Essen DC, Ugurbil K, Auerbach E, et al. The Human Connectome Project: a data acquisition perspective. Neuroimage 2012;62:2222–2231 doi: 10.1016/j.neuroimage.2012.02.018.
